# Development of a Dual Plasma Desorption/Ionization System for the Noncontact and Highly Sensitive Analysis of Surface Adhesive Compounds

**DOI:** 10.5702/massspectrometry.S0075

**Published:** 2017-12-08

**Authors:** Mari Aida, Takahiro Iwai, Yuki Okamoto, Satoshi Kohno, Ken Kakegawa, Hidekazu Miyahara, Yasuo Seto, Akitoshi Okino

**Affiliations:** 1 Laboratory for Future Interdisciplinary Research of Science and Technology, Tokyo Institute of Technology; 2 Department of Applied Chemistry for Environment, Kwansei Gakuin University; 3 FIRST, Institute of Innovative Research, Tokyo Institute of Technology; 4 National Research Institute of Police Science

**Keywords:** low-temperature plasma, desorption and ionization, mass spectrometry, atmospheric plasma

## Abstract

We developed a dual plasma desorption/ionization system using two plasmas for the semi-invasive analysis of compounds on heat-sensitive substrates such as skin. The first plasma was used for the desorption of the surface compounds, whereas the second was used for the ionization of the desorbed compounds. Using the two plasmas, each process can be optimized individually. A successful analysis of phenyl salicylate and 2-isopropylpyridine was achieved using the developed system. Furthermore, we showed that it was possible to detect the mass signals derived from a sample even at a distance 50 times greater than the distance from the position at which the samples were detached. In addition, to increase the intensity of the mass signal, 0%–0.02% (v/v) of hydrogen gas was added to the base gas generated in the ionizing plasma. We found that by optimizing the gas flow rate through the addition of a small amount of hydrogen gas, it was possible to obtain the intensity of the mass signal that was 45–824 times greater than that obtained without the addition of hydrogen gas.

## INTRODUCTION

Methods for semi-invasive and highly sensitive analysis of the compounds attached to the surface of heat-sensitive substances, such as skin, are necessary for various fields, such as medicine, manufacture of cosmetics, food hygiene, and criminal investigation.^1–4)^ For example, the trace amounts of chemical compounds contained in sweat or sebum on the skin may be used as indicators that reflect the physical condition of the body. The development of a highly sensitive analysis system for skin-surface compounds may enable the early diagnosis of diseases in the medical field.^5)^

Currently, various analytical methods have been proposed for analyzing surface compounds, and several devices have been developed. The wiping method^2–6)^ or direct analysis in real time (DART) developed by Cody *et al.*^7)^ are good examples of such methods. In the wiping method, after the surface compounds are wiped with a cotton swab or gauze, various analyses, such as organic or inorganic analyses,^8–10)^ are performed according to the type of the target compounds. Because the collected sample can be examined using various analytical methods,^11)^ it is thought that an increase in the number of degrees of freedom in the analysis can allow us to obtain highly reliable results.^9,12)^ However, because a pretreatment, such as extraction, is necessary for the sample, real-time analysis cannot be realized.

**Figure 1:**
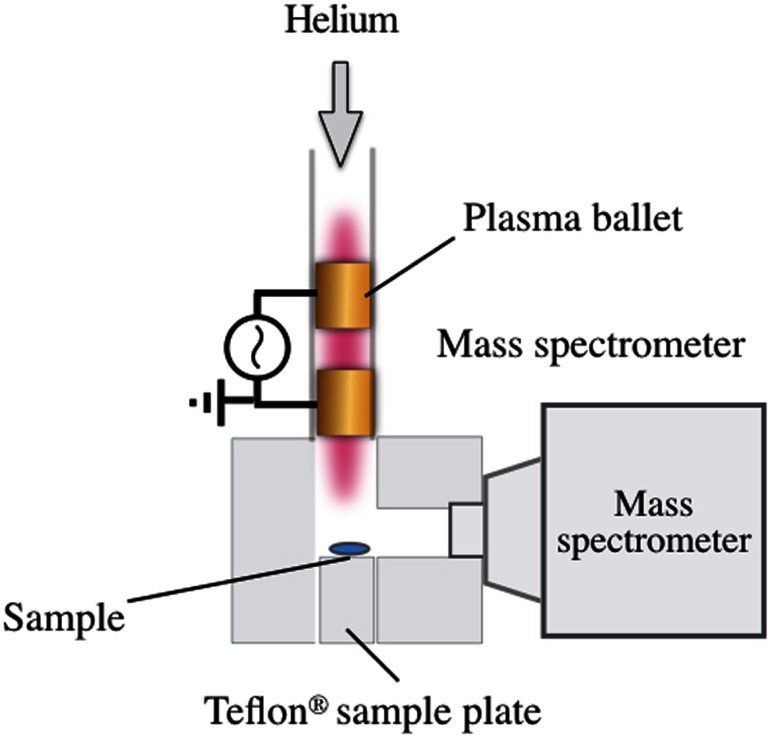
Fig. 1. Schematic of the atmospheric pressure plasma soft ablation (APSA) system with a low-temperature plasma source for performing desorption and ionization of the samples.

In the DART method, metastable helium atoms are generated by a DC glow discharge^13)^ between the electrodes through which the helium gas is flowed, and these metastable atoms are heated to a temperature of 250°C or higher using a heater and irradiated onto the sample. The sample is then analyzed by desorption and ionization. Even though no pretreatment of the sample is necessary in this method because the high-temperature gas is directly irradiated to the sample on the surface, it is difficult to apply this method to heat-sensitive surfaces, such as skin. In addition, since the desorption and ionization of sample are performed simultaneously, they cannot be analyzed at a given position, except by using a mass spectrometer, which has a device structure that is unfavorable for practical use. To overcome these challenges, the atmospheric pressure plasma soft ablation (APSA) method shown schematically in Fig. 1 was developed in our laboratory.^14–16)^ As the low-temperature plasma was used,^17,18)^ the sample can be analyzed without giving rise to heat damage to the surface.^19)^ In the APSA method, low-temperature plasmas generated with helium are used to perform desorption and ionization of the sample on the surface. First, when the low-temperature plasma is irradiated onto the compounds on the surface, these compounds are desorbed from the surface by the reaction with active species^20)^ generated in the plasma. Simultaneously, protons generated from the water molecules contained in the atmosphere or the helium bomb are applied to the desorbed samples. Subsequently, they are analyzed using a mass spectrometer. In this analytical method, plasma is irradiated only onto the compounds on the surface to be analyzed, enabling the analysis of samples in real time without any pretreatment. Additionally, the generated plasma is at approximately room temperature,^17,18)^ so it is possible to analyze surface compounds on heat-sensitive surfaces, such as plastic and fibers. This method was also used to analyze several pharmaceuticals and chemical warfare agents at the detection limits of several pmol.^16–21)^

Despite its advantages, the APSA method is still not suitable for practical applications because the desorption and ionization components of the system are required to be located close to the mass spectrometer, requiring the use of the instrument at a fixed and rather small distance from the sample. This is problematic due to the unwieldy nature of the mass spectrometer.

Therefore, we developed a dual plasma desorption/ionization system using two plasma sources for the desorption and ionization of the sample in order to realize an easy-to-use device structure while still obtaining a detection capability that is comparable to that of the conventional APSA system. In the conventional system, it was difficult to optimize each process because desorption and ionization were performed simultaneously by a single plasma source, whereas in the proposed system, two separate plasmas were used for desorption and ionization, making it possible to separate the desorption and ionization processes, and then separately optimize each process. Furthermore, since the ionization plasma is placed close to the mass spectrometer, the ionized sample can be quickly introduced into the mass spectrometer. This enables the more portable desorption component to be located far from the bulky and less portable mass spectrometer, making the system more suitable for practical use.

Additionally, the new dual plasma desorption/ionization system eliminates another problem caused in the conventional APSA system. In the conventional APSA method, protons generated from the moisture contained in the atmosphere or gas bomb are used for the ionization of the sample so that the amount of protons provided by the ionization plasma to the desorbed plasma fluctuates according to the changes in the ambient humidity and ionization efficiency.^22)^ For a reliable and highly sensitive analysis of various samples, it is necessary to control the proton amount.

To solve this problem, the dual plasma desorption/ionization system was designed such that it was possible to individually control the desorption and ionization components of the developed system. Furthermore, experiments were performed for improving the efficiency of the proton supply to the desorbed sample in the ionization component. We found that by mixing hydrogen gas into the ionization plasma gas, large amounts of stable protons were generated and the desorbed samples were efficiently ionized. Subsequently, the influence of proton amount on the mass signal intensity of sample was investigated.

## EXPERIMENTAL

### Dual plasma desorption/ionization system

**Figure 2:**
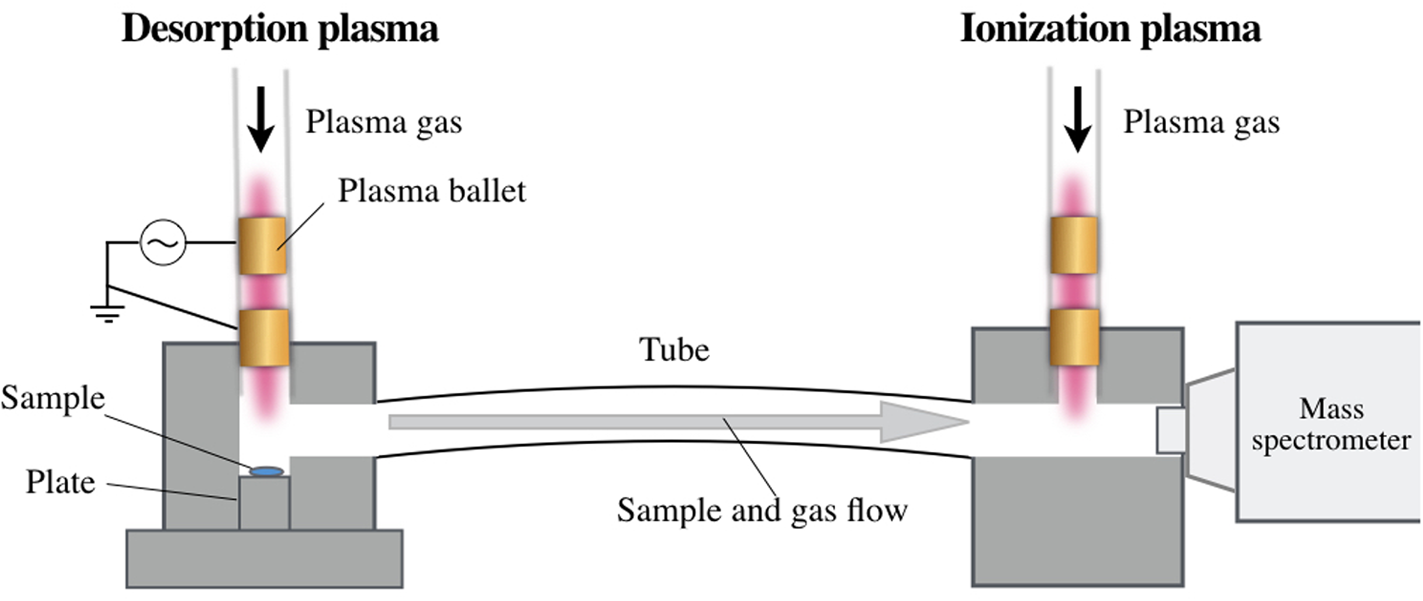
Fig. 2. Schematic of the newly developed system with individual plasma sources for desorption and ionization of the adsorbed sample.

A schematic of the developed system is shown in Fig. 2, which describes the entire process until the sample is mass analyzed. First, the sample on the sample plate placed just under the desorption plasma source is irradiated by the desorption plasma. Then, the sample is desorbed by the active species generated in the plasma. Further, the desorbed sample is drawn using the plasma gas and the negative pressure of the mass spectrometer so that it passes through the transport tube and is transported to the ionization component. Subsequently, the desorbed sample is irradiated with the ionization plasma and the protons generated from the gas bomb or moisture in the system are applied to the desorbed sample, followed by ionization. Finally, the inlet of the mass spectrometer is directly connected to the transport tube and the ionized sample is introduced into the mass spectrometer. All processes from the desorption of the sample to the introduction into the mass spectrometer are performed in a closed space.

Sample on plate is irradiated by desorption plasma and then the sample is desorbed. Desorbed sample passes through the transport tube. The desorbed sample is irradiated by ionization plasma, and then it is ionized. The ionized sample is introduced into the mass spectrometer.

In the experiments performed in this study, two mass spectrometers were used because the experiments were conducted in our laboratory and at the National Research Institute of Police Science (NRIPS). In our laboratory, the amaZon-SL-AI ion trap mass spectrometer (referred to as MS1 in this study; Bruker Daltonics K.K.) was used, whereas at the NRIPS, the 1100 Series LC/MSD ion trap mass spectrometer (referred to as MS2 in this study; Agilent Technologies) was used. The design of the produced plasma source differs somewhat for the two spectrometers due to the differences in the conditions for the stabilization of the plasma source and the sensitivity. When MS1 was used, a glass tube (Pylex made; outer diameter=5 mm and inner diameter=3 mm) was used as the plasma source, a 10-mm-wide copper tape was wound around the external portion of the glass tube at a position 10 mm from the blowout port of the plasma, and another 10-mm-wide copper tape was wound around the external portion of the glass tube at an interval of 5 mm. We used a Teflon^®^ tube (outer diameter=3 mm and inner diameter=1 mm) that functioned as a transport tube connecting both plasma sources. In the mass spectrometer, the capillary voltage was changed from −1000 to −6000 V and the integration time was varied from 10 to 100 ms. When MS2 was used, a glass tube (Pylex made; outer diameter=6 mm and inner diameter=4 mm) was used as the plasma source, a 10-mm-wide copper tape was wound around the external portion of the glass tube at a position 10 mm from the blowout port of the plasma, and another 10-mm-wide copper tape was wound around the external portion of the glass tube at an interval of 10 mm. Furthermore, in this case, we used a Teflon^®^ tube (outer diameter=6 mm and inner diameter=4 mm) that functioned as a transport tube connecting both plasma sources. For the sample plate irradiated by desorption plasma, a Teflon^®^ rod was excavated to a diameter of 3 mm and a depth of 1 mm at the tip; the sample was placed on this area. In the mass spectrometer, the capillary voltage was set to be −500 V and the integration time was varied up to 100 ms. When both MS1 and MS2 were used, argon gas was used as a plasma gas between the electrodes of the developed plasma; this plasma was maintained by an alternating current power source with a voltage of 9 kV and a frequency of 15 kHz. The electrode on the side from which the plasma of the desorption plasma source is blown off was grounded so that the sample on the surface is desorbed without any discharge damage.

### Sample preparation

As the samples, the following ingredients used for the pharmaceutical products and products in daily use were selected: 2-isopropylpyridine was used for dyeing and stain removal, isopropylantipyline was used for analgesics, *o*-ethoxybenzamide was used for antipyretic analgesics, and 4-isopropylaniline was used for herbicides. The characteristics of each sample are listed in Table 1.

**Table 1:** Table 1. Chemical structures, molecular weights, target ions.

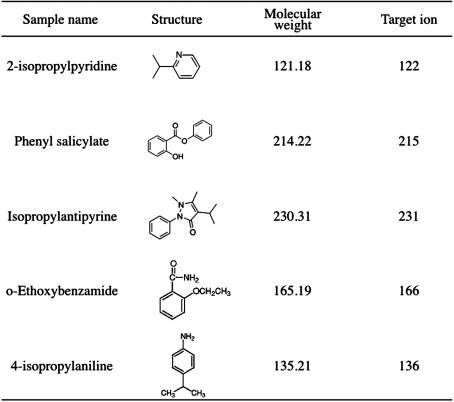

First, each sample was dissolved in methanol (Wako Pure Chemical Industries, Ltd., Osaka, Japan); then, 5 μL of the solution was sampled, dropped on the sample plate, and allowed to stand until the solvent dried. The absolute amounts after drying was 5 ng for phenyl salicylate, 50 pg for 2-isopropylpyridine, and 0.5 ng for all other samples.

## RESULTS AND DISCUSSION

### —Mass spectrometry of the sample and evaluation—

#### Analysis using the conventional APSA method

**Figure 3:**
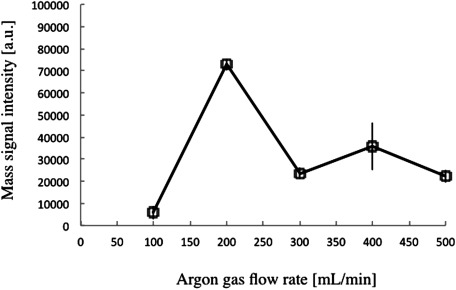
Fig. 3. Maximum signal intensity of phenyl salicylate (*m*/*z*=215) as a function of argon gas flow rate.

To demonstrate the advantages of the developed system, an experiment was performed using the conventional APSA method wherein desorption and ionization were performed with one plasma source, which allowed a comparison with the results obtained using the new dual plasma system. The position of the plasma source was set at 30 mm from the tip of the capillary, and argon gas was used as the plasma gas. The condition of the plasma source and the organic mass spectrometer were the same as those described for MS1 in the experimental section. Phenyl salicylate was used as the sample. The results of the relation between the argon gas flow rate used for the plasma gas and the signal intensity of the ion (*m*/*z*=215 ([C_13_H_10_O_3_]H^+^)) derived from the sample are shown in Fig. 3. It was confirmed that the signal intensity of ion (*m/z*=215) was maximum when the argon gas flow rate was 200 mL/min.

#### Analysis using the dual plasma desorption/ionization system

For the dual plasma desorption/ionization system developed in this study, the length of the transport tube connecting the desorption and ionization plasmas was changed between 300 and 500 mm and phenyl salicylate was examined in a manner similar to that of the conventional method. First, when only the desorption plasma was irradiated, we were unable to detect any mass spectral signals of each sample for the tube lengths of 300 and 500 mm, even if the plasma gas was changed. This is because even though the sample was desorbed by plasma irradiation, the distance to the mass spectrometer was so long that the sample was not sufficiently ionized.

On the other hand, when both the desorption and ionization plasmas were irradiated simultaneously, for the 300-mm-long transport tube, desorption plasma gas of 600 mL/min, and ionization plasma gas of 400 mL/min, a mass spectrum, *m*/*z*=[C_13_H_10_O_3_]H^+^, derived from the sample was confirmed. For the 500-mm-long transport tube, desorption plasma gas of 600 mL/min, and ionization plasma gas of 200 mL/min, in a mass spectrum, ion (*m*/*z*=215) derived from the sample and the fragment ion (*m*/*z*=121 (C_6_H_4_(OH)CO^+^)) was observed.

**Figure 4:**
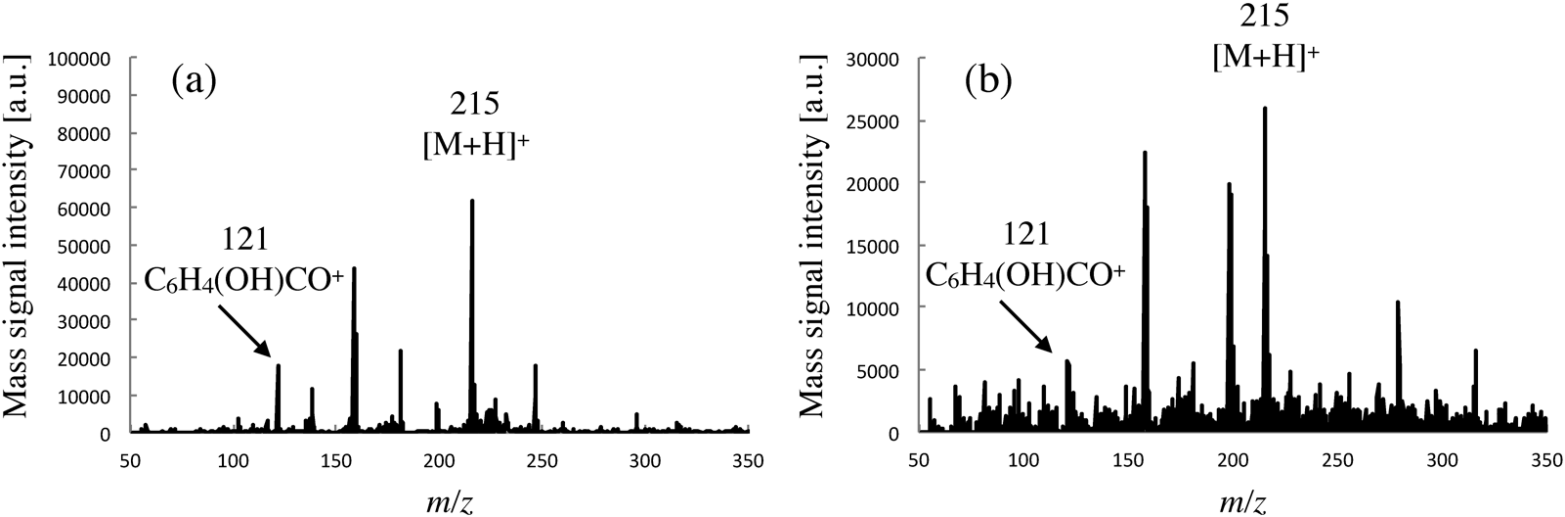
Fig. 4. Mass spectrometric analysis of phenyl salicylate with a dual plasma system equipped with (a) 300-mm long and (b) 500-mm long transport tubes.

The relation between *m*/*z* and mass signal intensity obtained under these conditions is shown in Fig. 4.

**Figure 5:**
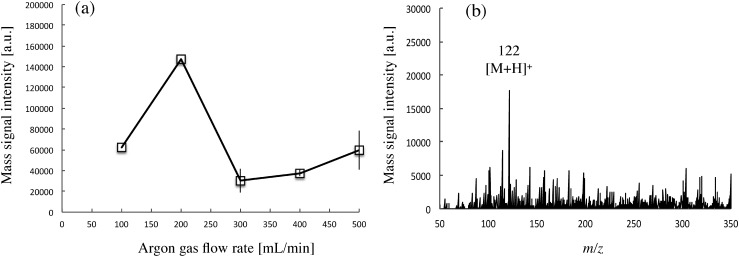
Fig. 5. Mass spectrometric analysis of 2-isopropylpyridine dual plasma system.

A decrease in the signal intensity was observed as the position where the sample was desorbed moved away from the mass spectrometer. However, since the protons were applied to the desorbed sample in the ionization component in front of the mass spectrometer inlet, the ion signals derived from the sample was detected even for long distances between the desorption and ionization components. In addition, as shown in Fig. 4(a), the sum of the mass signal intensity of the ion (*m*/*z*=215 ([C_13_H_10_O_3_]H^+^)) and the fragment ion (*m*/*z*=122 (C_6_H_4_(OH)CO^+^)) was comparable to the mass signal intensity obtained using the conventional APSA method under an argon gas flow rate of 200 mL/min. Therefore, it is quite likely that a part of the desorbed sample was fragmented by the ionization plasma. Furthermore, as shown in Fig. 4(b), due to the long transport tube, it is considered that the desorbed sample transported through the tube was adsorbed on the internal tube. Therefore, the amount of the desorbed sample transported to the ionization component decreased, leading to a corresponding decrease in the mass signal intensity at *m*/*z*=215 ([C_13_H_10_O_3_]H^+^). Next, 2-isopropylpyridine was used as the sample and the same experiment was conducted. The mass spectrum at *m*/*z*=122 ([C_8_H_11_N]H^+^) derived from the sample was observed for the analysis at the distance of the transport tube of 500 mm, the desorption plasma gas of 300 mL/min, and the ionization plasma gas of 600 mL/min. The results for 2-isopropylpyridine are shown in Fig. 5. These experiments show that the developed system is suitable for practical applications.

(a) The signal intensity of protonated 2-isopropylpyridine (*m*/*z*=122) as a function of the argon gas flow rate. (b) The mass spectrum of 2-isopropylpyridine taken with a 500-mm length transport tube.

### —Toward highly sensitive analysis of samples—

#### Water removal in gas bomb and emission spectroscopic analysis

As mentioned earlier, in the conventional APSA method, the protons generated from the moisture contained in the atmosphere or from the gas bomb were used for the ionization of the sample. This presents a problem wherein the ionization efficiency of the protons applied to the desorbed sample fluctuates with the changes in the ambient humidity. This effect can significantly lower the detection sensitivity and must be controlled for realizing practical application of the APSA method to the analyses of various samples.

In the conventional APSA method, because the desorption and ionization of sample were performed with a single plasma source, both processes cannot be optimized individually. Therefore, it appears that there is a limit to achieve an improvement in the sensitivity of the system from the standpoint of the plasma generation conditions. In contrast, in the new dual plasma system, because the desorption and ionization components can be individually controlled, it is possible to freely change the plasma generation conditions used in each process.

**Figure 6:**
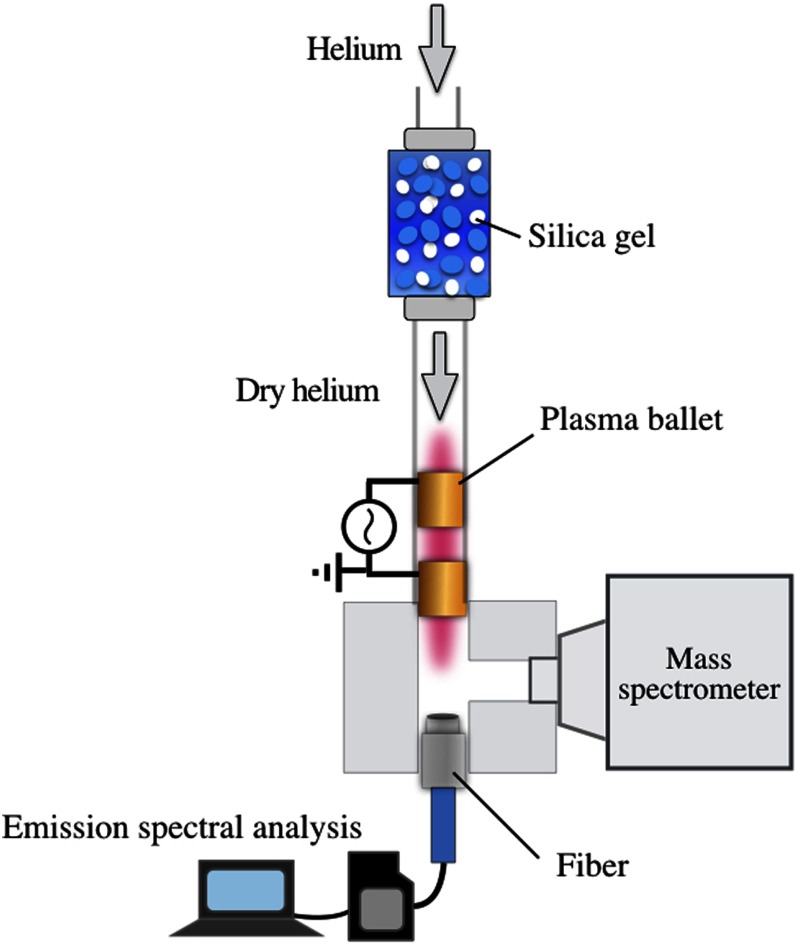
Fig. 6. Emission spectroscopic analysis of the hydrogen atom H_α_.

Exploiting this advantage, a new method was proposed for supplying the protons by the addition of hydrogen to the base gas generated by the ionization plasma, unlike the moisture which cannot be easily controlled in the atmosphere or in a gas bomb. First, a trace amount of the moisture contained in the gas supplied from the gas bomb to the plasma was removed by passing through a silica gel, as illustrated in Fig. 6. Considering that the excitation energies of hydrogen (12.7 eV) and helium (19.8 eV) were larger than the excitation energy (11.6 eV) of the metastable state of argon^22)^ used for the plasma gas, the gas flow rate was changed from 50 to 500 mL/min.

**Figure 7:**
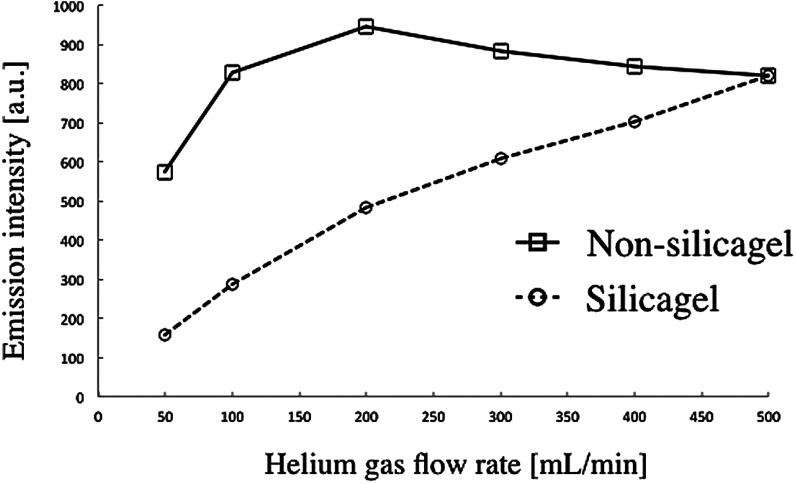
Fig. 7. Intensity of the H_α_ emission of helium plasma as a function of the helium gas flow rate.

Generally, the emission spectral peaks of hydrogen atoms, such as H_α_ (656.28 nm) or H_β_ (486.13 nm), can be observed *via* emission spectroscopic analysis of the plasma containing moisture.^22,23)^ In this experiment, to confirm the usefulness of the moisture removal by the silica gel, the emission intensities of H_α_ were observed from the axial direction from the plasma outlet when the plasma was generated by passing the plasma gas through a silica gel. And when plasma gas was supplied directly from the gas bomb to the plasma source; moreover the amount of the moisture was evaluated; the results of these evaluations are shown in Fig. 7. The observed emission spectral peaks of hydrogen is considered to be due to the hydrogen atoms generated from the trace amount of moisture present in the helium bomb supplied to the system. Except for the case of the helium gas flow rate of 500 mL/min, the emission intensity of atomic hydrogen was decreased by passing through the silica gel. In particular, it was confirmed that the hydrogen atom emission intensity decreased by 73% at a helium gas flow rate of 50 mL/min and that the silica gel was effective for the removal of the moisture.

### Mass spectrometry of sample with hydrogen addition

**Figure 8:**
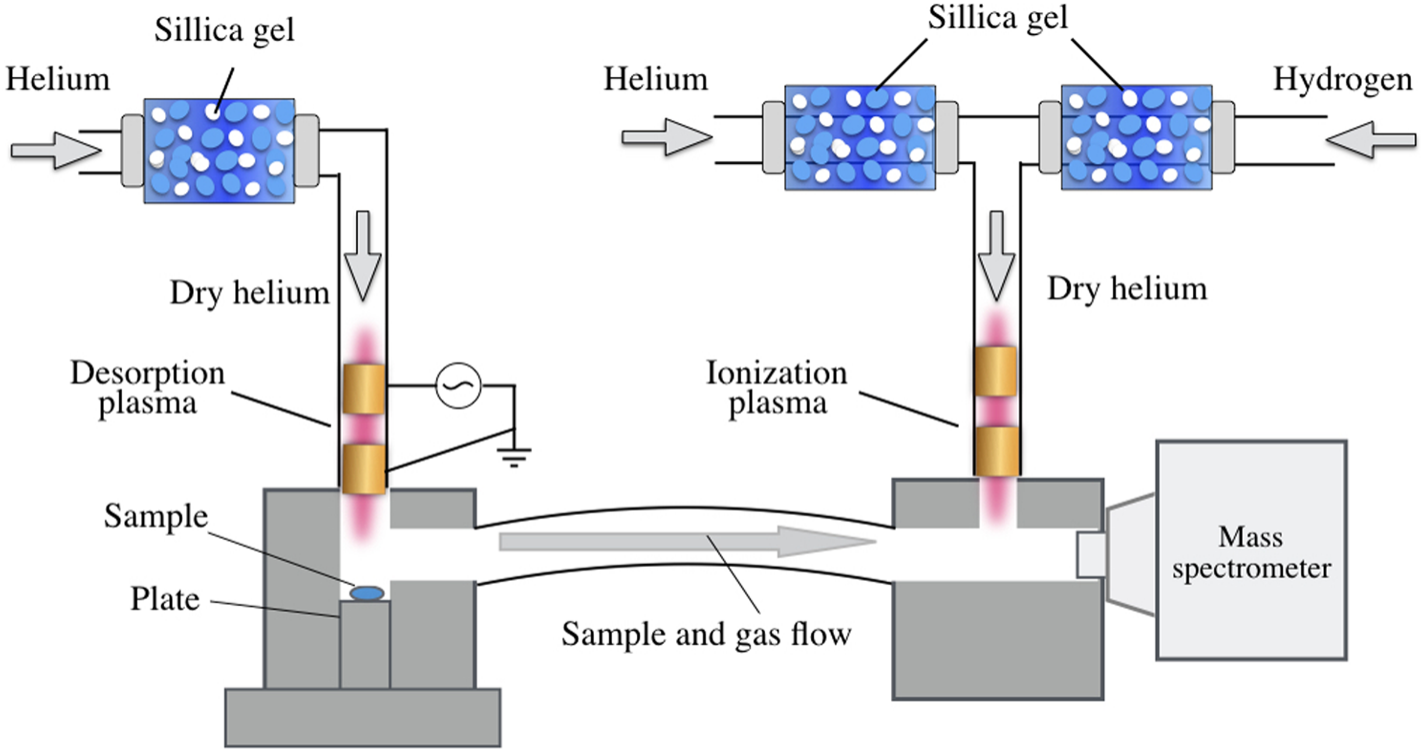
Fig. 8. Mass spectrometry performed *via* hydrogen.

As shown in Fig. 8, dry helium passing through the silica gel was also used for the desorption plasma in order to prevent contamination by different gas species and the moisture supplied into the system through desorption plasma. The helium flow rates of 200 and 50 mL/min for the desorption and ionization plasmas, respectively, were used in the experiments. Furthermore, the protons generated in the plasma were used for the ionization of the sample by adding 0%–0.02% of hydrogen to helium in the ionization plasma. The length of the transport tube was 100 mm. Isopropylantipyrine, 2-isopropylpyridine, and 4-isopropylaniline were used as the samples and analyzed under the conditions described in the Experimental section for MS1. Furthermore, under the conditions described for MS2, isopropylantipyrine and *o*-ethoxybenzamide were used as the samples and the same experiment was conducted.

A trace amount of the moisture contained in helium gas and hydrogen gas introduced into each plasma are removed by the silica gel. The sample on the plate is irradiated by desorption plasma and then desorbed. The desorbed sample passes through the transport tube. It is first irradiated by ionization plasma and then ionized by the proton derived from hydrogen. The ionized sample is introduced into the mass spectrometer.

**Figure 9:**
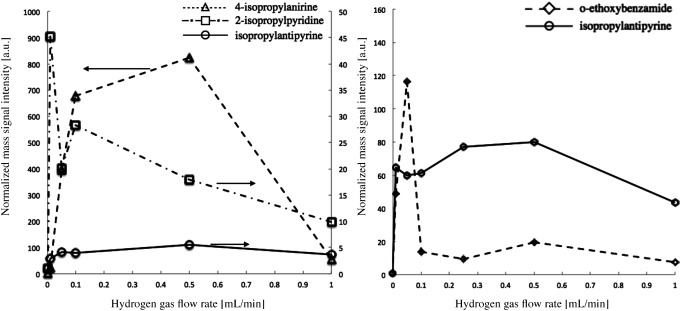
Fig. 9. (a) Relation between the hydrogen gas flow rate added to helium and normalized signal intensity in isopropylantipyrine, 2-isopropylpyridine, and 4-isopropylanirine, determined using an amazon-SL-AI ion trap mass spectrometer (MSI). The results were improved by 6, 45, and 824 times when the hydrogen gas was added to the helium base gas at flow rates of 0.5, 0.01, and 0.5 mL/min, respectively. (b) Relation between the hydrogen gas flow rate added to helium and normalized signal intensity in isopropylantipyrine and *o*-ethoxybenzamide, determined using the Agilent 1100 Series LC/MSD ion trap mass spectrometer (MS2). The results were improved by 80 and 117 times when the hydrogen gas was added to the helium base gas at flow rates of 0.5 and 0.05 mL/min, respectively.

The relation obtained between the gas flow rate of hydrogenation and the signal intensity is shown in Fig. 9. Here, the mass signal intensities were normalized to the signal intensity in the absence of added hydrogen. When isopropylantipyrine, 2-isopropylpyridine, and 4-isopropylaniline were used as the samples under the conditions described for MS1, improvements by a factor of 6, 45, and 824 were obtained for the hydrogen gas flow rates of 0.5, 0.01, and 0.5 mL/min, respectively. In contrast, when isopropylantipyrine and *o*-ethoxybenzamide were used as the samples under the conditions described for MS2, improvements by a factor of 80 and 117 for the hydrogen gas flow rates of 0.5 and 0.05 mL/min, respectively, were obtained. Increases and decreases in the signal intensity were observed when the hydrogen gas flow rates were varied. When isopropylantipyrine was used as the sample, similar changes associated with mass signal intensity were observed in the experimental systems MS1 and MS2. For example, the volatilities of isopropylantipyrine, 2-isopropylpyridine, 4-isopropylaniline are in the order of isopropylantipyrine <2-isopropylpyridine <4-isopropylanirine and the rate of change in the signal intensity also increases in this order. Because the gas temperature of the plasma used in the system is sufficiently high for application to living bodies, it is unlikely that the results of the analysis are influenced by heat. Nevertheless, in the future, it is essential to investigate the effect of small temperature change of plasma gas.

As mentioned above, by adding hydrogen to the ionization plasma and optimizing the gas flow rate, it is possible to improve the ionization efficiency of the sample and increase the mass signal intensity, even for the samples with different properties, demonstrating the advantages and usefulness of the proposed dual plasma method.

## CONCLUSION

In this research, to develop a more practical system with improved analytical performance, a dual plasma desorption/ionization system with two plasma sources was developed. In this system, it was possible to individually optimize the desorption plasma of the sample and the ionization plasma used for the ionization of the desorbed sample. In addition, the ionization plasma source was placed close to the inlet of the mass spectrometer and the ionized sample was quickly introduced into the mass spectrometer, thereby enabling the use of the desorption component at a distance from the mass spectrometer. This provided a system structure suitable for use in practical applications.

Using the developed system, phenyl salicylate with low-volatility and 2-isopropylpyridine with relatively high volatility were examined. Even when the desorption and ionization plasmas were separated by 500 mm, it was possible to detect the mass spectral signals derived from each sample at a resolution similar to that for the conventional APSA system by individually optimizing the gas flow rates of both plasmas. This results in an increase in the distance between the sample position and the mass spectrometer by a factor of more than 50, demonstrating the usefulness of the developed method and the practicality of the system.

Furthermore, since the desorption and ionization of the sample can be controlled individually, the ionization process was primarily investigated and experiments were conducted to improve the ionization efficiency of the desorbed sample. A small amount of hydrogen was added to the helium base gas that generates the ionization plasma at an optimized gas flow rate, enabling control of the amount of the protons necessary for the ionization of sample. As a result, for the analysis of isopropylantipyrine, 2-isopropylpyridine, 4-isopropylanirine, and *o*-ethoxybenzamide, the obtained signal intensities were 45 to 824 times greater than those obtained without hydrogenation.

Our future research will focus on the analysis of the samples required for various onsite analyses or contaminant identification and the study of high sensitivity and practical improvement of the system.

## Notes

Mass Spectrom (Tokyo) 2017; 6(3): S0075
